# Cell-type specific connectivity accounts for diverse in vivo functional roles of inhibitory neurons in V1

**DOI:** 10.1186/1471-2202-16-S1-P165

**Published:** 2015-12-18

**Authors:** Jung H Lee, Stefan Mihalas

**Affiliations:** 1 Allen Institute for Brain Science, Seattle, WA 98103, USA

## 

Inhibitory neurons have a large diversity [1], however functional roles of diverse subtypes have not been elucidated. We aim to help systematically addressing this question using large scale modeling and coarse graining between scales. Mesoscopic models of in vivo activity starting from structure have only recently become feasible making use of development of cortical column models [2], knowledge of connectivity [3,4], scalable simulation tools [NEST] and robust population statistic techniques [5].

**Figure 1:**
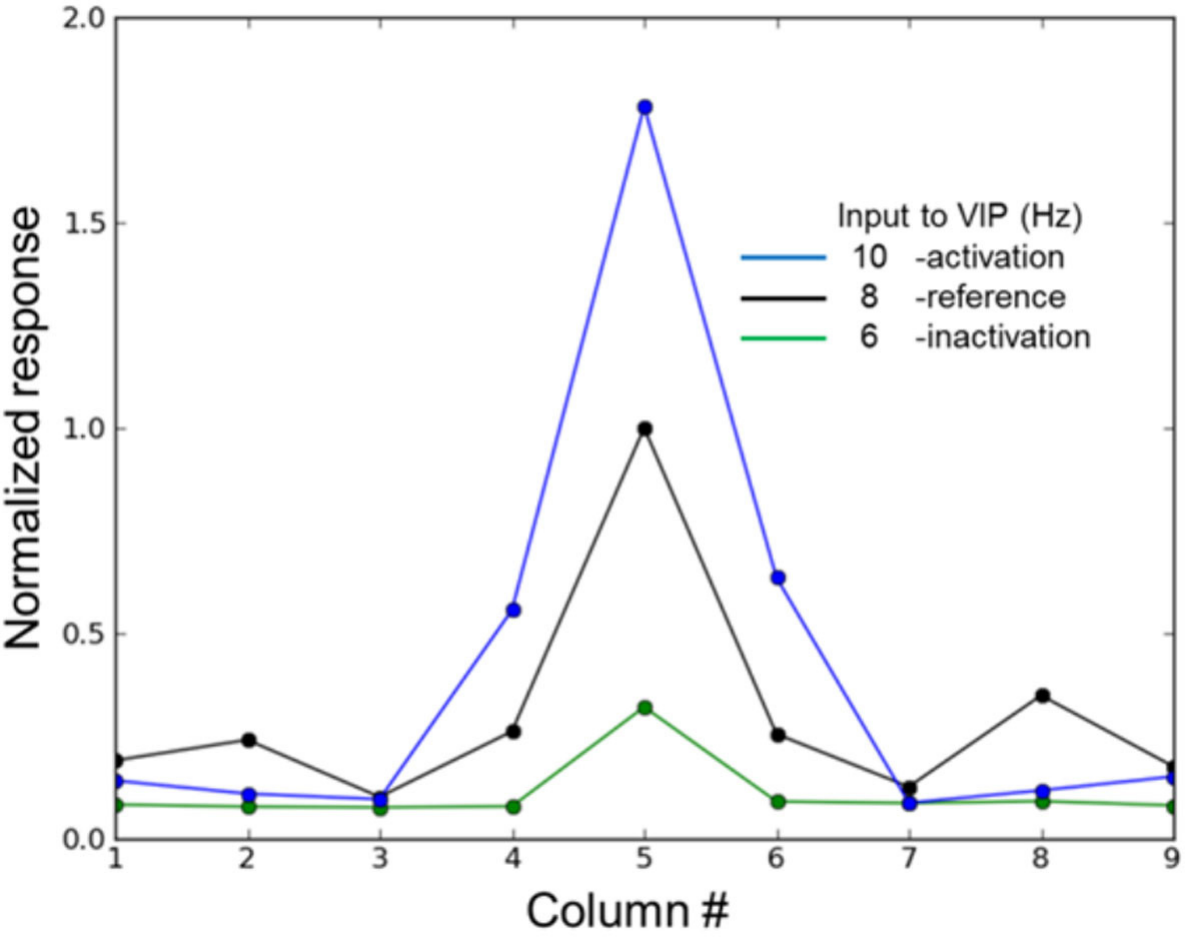
**Simulated tuning curve for pyramidal neurons as a function of top-down inputs to VIP cells**. These inputs allow a multiplicative gain modulation of the bottom-up input.

Vasoactive intestinal peptide-positive (VIP+) interneurons are one of the major inhibitory cell types in the cortex [1], and their excitability has been found to be associated with various endogenous factors such as brain states and top-down signals. Zhang et al. [6] recently found that top-down signals originated in the cingulate (Cg) strongly innervate VIP + interneurons of V1, indicating the critical role of VIP+ cells in attentional gain modulation. We studied their effect on top-down signals using both analytical and computational models. Using a coarse-grained firing rate model for the cell types in the superficial layers we found that the reported cell-type specific connectivity allows top-down inputs to VIP+ interneurons to effectively disinhibit pyramidal cell activity. Due to the limitations of the firing rate model, we utilized large scale computational models to investigate mechanisms underlying attentional gain modulation in an entire network. Specifically, we adopted the multiple column model proposed by Wagatsuma et al. [4] and modified them by incorporating three inhibitory cell types into the superficial layers of the model. In our model we considered parvalbumin-positive (PV+), SST+ and VIP+ inhibitory cells. We also constructed the data-driven cell-type specific intercolumnar connections between columns. Our model was capable of reproducing multiplicative gain modulation (see Figure 1), and our simulation results suggest that non-specific activation of VIP cells is sufficient for generating multiplicative gain modulation.

We also note that PV+ cell-mediating inhibition across columns is critical for regulating the responses over small spatial scales: e.g. induced by non-preferred stimulus (orientation). **SST+ cell mediate inhibition across columns and allow contextual visual processing in V1**.
